# The inverse Wiener polarity index problem for chemical trees

**DOI:** 10.1371/journal.pone.0197142

**Published:** 2018-05-11

**Authors:** Zhibin Du, Akbar Ali

**Affiliations:** 1 School of Mathematics and Statistics, Zhaoqing University, Zhaoqing 526061, Guangdong, P.R. China; 2 Knowledge Unit of Science, University of Management & Technology, Sialkot, Pakistan; University of Lincoln, UNITED KINGDOM

## Abstract

The Wiener polarity number (which, nowadays, known as the Wiener polarity index and usually denoted by *W*_*p*_) was devised by the chemist Harold Wiener, for predicting the boiling points of alkanes. The index *W*_*p*_ of chemical trees (chemical graphs representing alkanes) is defined as the number of unordered pairs of vertices (carbon atoms) at distance 3. The inverse problems based on some well-known topological indices have already been addressed in the literature. The solution of such inverse problems may be helpful in speeding up the discovery of lead compounds having the desired properties. This paper is devoted to solving a stronger version of the inverse problem based on Wiener polarity index for chemical trees. More precisely, it is proved that for every integer *t* ∈ {*n* − 3, *n* − 2,…,3*n* − 16, 3*n* − 15}, *n* ≥ 6, there exists an *n*-vertex chemical tree *T* such that *W*_*p*_(*T*) = *t*.

## Introduction

A (chemical) topological index is a real number calculated from chemical graphs (graphs representing chemical compounds, in which vertices represent atoms and edges represent covalent bonds between atoms) such that it remains unchanged under graph isomorphism [1]. Topological indices are usually used in quantitative structure-activity and structure-property relationships studies for predicting the biological activities or physical-chemical properties of chemical compounds [2].

The Wiener polarity number (which, nowadays, known as the Wiener polarity index and usually denoted by *W*_*p*_) was devised in 1947 by the chemist Harold Wiener [3] for predicting the boiling points of alkanes, and this index is among the oldest topological indices. The index *W*_*p*_ of chemical trees (chemical graphs representing alkanes) is defined as the number of unordered pairs of vertices at distance 3.

Lukovits and Linert [4] extended the definition of *W*_*p*_ for cycle-containing chemical graphs by using a heuristic approach, and used this new definition to demonstrate quantitative structure-property relationships in a series of acyclic and cycle-containing hydrocarbons. Hosoya and Gao [5] found that the relative magnitude of *W*_*p*_ among isomeric alkanes keeps pace with the number of gauche structures in the most probable confirmation, and thus *W*_*p*_ can predict the relative magnitude of liquid density. Miličević and Nikolić [6] used *W*_*p*_ in modeling the boiling points of lower (*C*_3_–*C*_8_) alkanes. Shafiei and Saeidifar [7] performed quantitative structure-activity relationships studies on 41 sulfonamides for predicting their heat capacity and entropy, using *W*_*p*_ together with some other topological indices. In a recent study [8], some models for predicting the thermal energy, heat capacity and entropy of 19 amino acids were developed and it was found that *W*_*p*_ is a good topological index for modeling thermal energy.

In the last decade, *W*_*p*_ has attracted a considerable attention from researchers, for example, see the recent papers [9–14] and related references listed therein.

In this paper, we are concerned with the possible values of *W*_*p*_ for chemical trees. As usual, denote by *uv* the edge connecting the vertices *u*, *v* in a chemical tree *T*, and *d*_*T*_(*u*) the degree of vertex *u* in *T*. The following beautiful result is due to Du *et al*. [15]:

**Lemma 1**. *Let T be a (chemical) tree. Then*

$$W_{p}\left(T\right)=\sum_{uv\in E(T)}\left(d_{T}\left(u\right)-1\right)\left(d_{T}\left(v\right)-1\right),$$

*where E*(*T*) *denotes the edge set of T*.

Here, it should be mentioned that *W*_*p*_ is the same as the reduced second Zagreb index [16–18] in case of (chemical) trees. Deng [19] reported the maximum *W*_*p*_ value of chemical trees. The same authors of this paper [20] characterized all the chemical trees with maximum *W*_*p*_ value. Recently, Ashrafi and Ghalavand [21] determined the first two minimum *W*_*p*_ values of chemical trees and characterized the corresponding chemical trees attaining the first two minimum *W*_*p*_ values. In the reference [22], main extremal results of the paper [21] are re-established in an alternative but shorter way, and all members with the third minimum *W*_*p*_ value are determined from the collection of all *n*-vertex chemical trees.

The problem of finding chemical structure(s) corresponding to a given value of a topological index TI is known as the inverse problem based on TI [23]. Solutions of such inverse problems may be helpful in designing a new combinatorial library, and speed up the discovery of lead compounds with some desired properties [24].

Study of the inverse problem based on topological indices was initiated by the Zefirov group in Moscow [25–29]. Gutman [30] studied the inverse problem based on the Wiener index (this index appeared in the same paper [3] where *W*_*p*_ was reported, see the recent survey [31] for more details about Wiener index). Solving the inverse problem based on Wiener index was the subject of several papers, for example see the papers [32–34] and related references listed therein. Li *et al*. [35] addressed the inverse problem based on four other well-known topological indices, introduced in mathematical chemistry. Recently, an inverse problem based on the *k*-th Steiner Wiener index (a generalized version of Wiener index) was studied in the paper [36]. Further details about inverse problem can be found in the survey [37], recent papers [38, 39] and related references listed therein.

Here we attempt to solve a stronger version of the inverse problem based on Wiener polarity index for chemical trees. We have been able to show that for every integer *t* ∈ {*n* − 3, *n* − 2,…,3*n* − 16, 3*n* − 15}, where *n* ≥ 6, there exists an *n*-vertex chemical tree *T* such that *W*_*p*_(*T*) = *t*.

## Methods

By Lemma 1, we may get the following two lemmas immediately.

**Fig 1:**
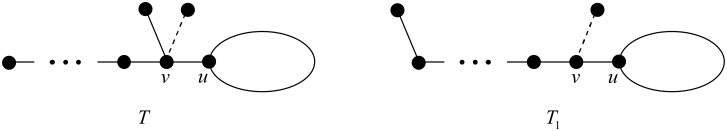
The chemical trees *T* and *T*_1_ in Lemma 2. (The edges which are represented by dashed lines may or may not occur in the tree).

**Lemma 2**. *Let T and T*_1_ *be the two chemical trees as depicted in* Fig 1. *Then*

$$W_{p}\left(T_{1}\right)-W_{p}\left(T\right)=-d_{T}\left(u\right)+1.$$

In particular, the transformation from *T* to *T*_1_ depicted in Lemma 2 is called a grafting pendent path transformation at *v* in *T*.

A vertex of degree 1 is said to be a pendent vertex.

**Lemma 3**. *Suppose that v is a pendent vertex with unique neighbor u in the chemical tree T*. *Let T*_1_ *be another chemical tree obtained from T by attaching a pendent vertex to v. Then*

$$W_{p}\left(T_{1}\right)-W_{p}\left(T\right)=d_{T}\left(u\right)-1.$$

## Results

**Theorem 1**. *For every integer n* − 3 ≤ *t* ≤ 3*n* − 15, *where n* ≥ 6, *there exists a chemical tree T of order n such that W_*p*_*(*T*) = *t, i.e*.,

$$\{W_{p}\left(T\right):T\;is\;a\;chemical\;tree\;of\;order\;n\}=\left\{n-3,n-2,\ldots ,3n-16,3n-15\right\}.$$

**Fig 2:**
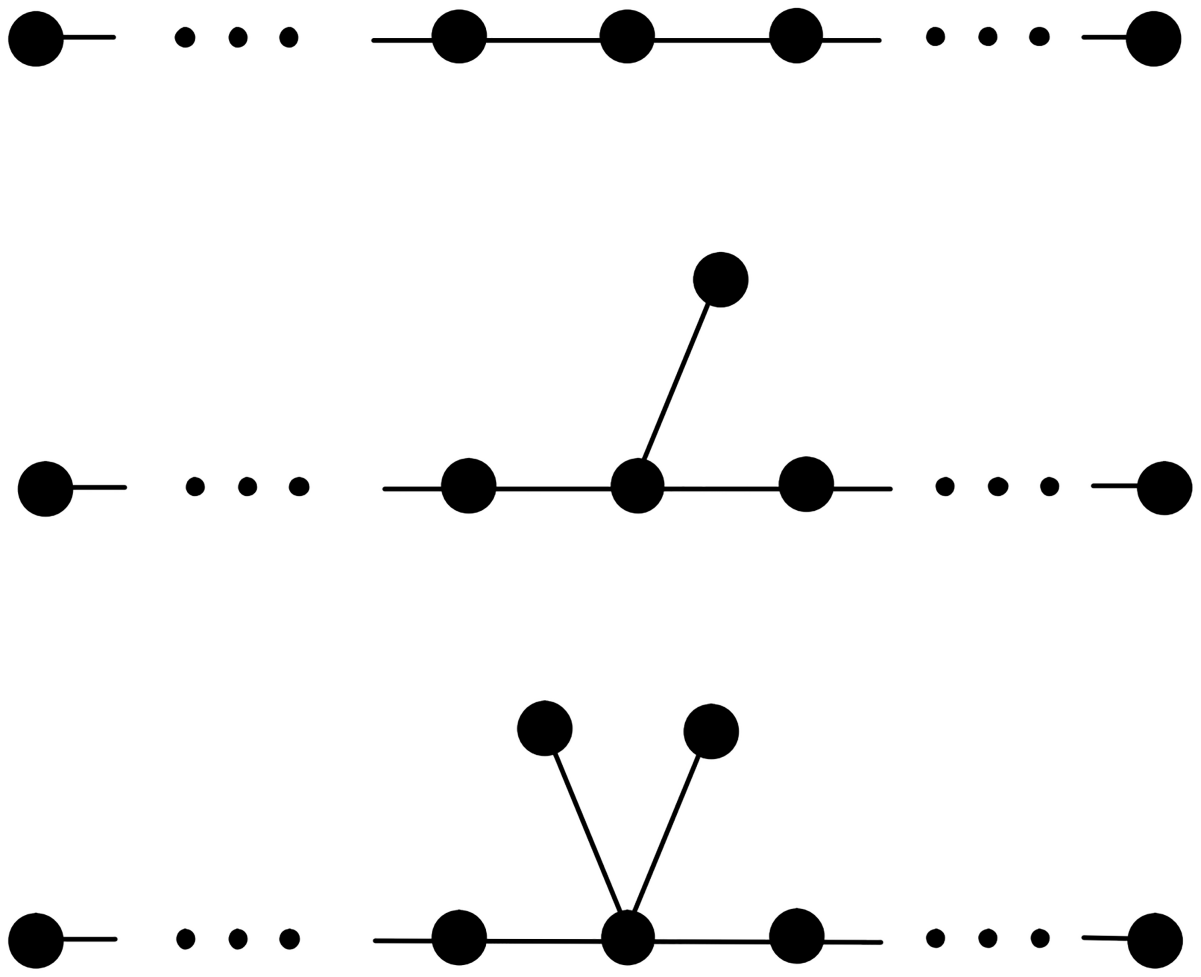
The chemical trees of order *n* with Wiener polarity indices *n* − 3, *n* − 2 and *n* − 1, respectively.

*Proof*. Clearly, the three chemical trees of order *n* depicted in Fig 2 have Wiener polarity indices *n* − 3, *n* − 2 and *n* − 1, respectively. So we need only to focus on the existence of chemical trees *T* of order *n* with *W*_*p*_(*T*) = *t*, where *n* ≤ *t* ≤ 3*n* − 15, i.e., *n* ≥ 8.

For the case *n* = 8, *t* can only be 8 or 9. It is easily checked that the chemical tree of order 8 obtained from *P* = *v*_1_*v*_2_*v*_3_*v*_4_*v*_5_ by attaching three pendent vertices each to *v*_2_, *v*_3_, *v*_4_ has Wiener polarity index 8, and the chemical tree of order 8 obtained from *P* = *v*_1_*v*_2_*v*_3_*v*_4_*v*_5_ by attaching a pendent vertex to *v*_2_ and two pendent vertices to *v*_3_ has Wiener polarity index 9.

Suppose in the following that *n* ≥ 9. We partition our proof into three cases according to the value *n*(mod 3).

**Case 1**. *n* = 3*k*, where *k* ≥ 3.

Since the results for *t* = *n* − 3, *n* − 2, *n* − 1, or equivalently, *t* = 3*k* − 3, 3*k* − 2, 3*k* − 1, follow from Fig 2, we are left to consider *n* ≤ *t* ≤ 3*n* − 15, which is equivalent to 3*k* ≤ *t* ≤ 9*k* − 15.

**Fig 3:**
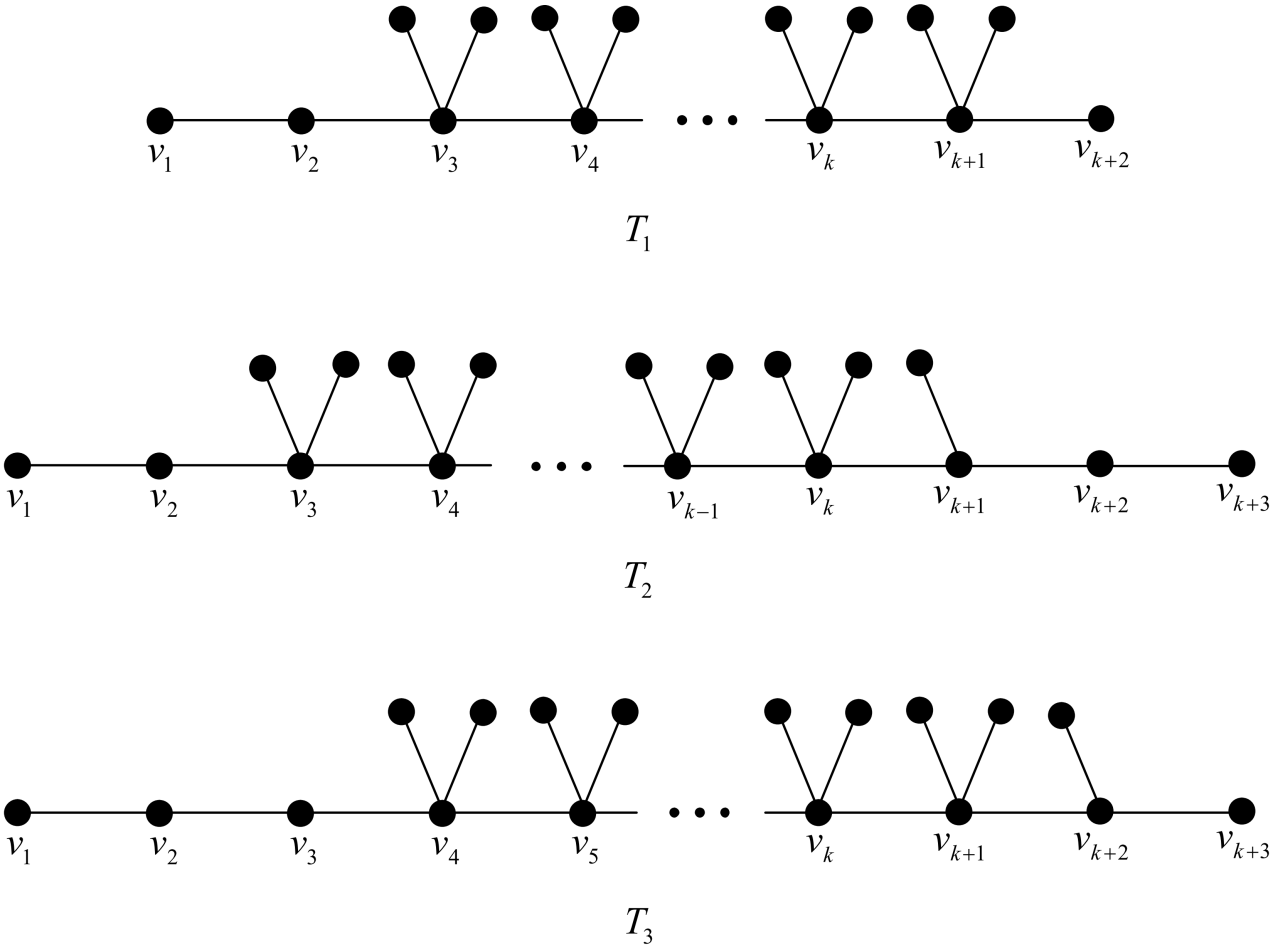
The chemical trees *T*_1_, *T*_2_ and *T*_3_ in the proof of Case 1 in Theorem 1.

For the three chemical trees *T*_1_, *T*_2_ and *T*_3_ of order *n* = 3*k* in Fig 3, it is easily verified that

$$W_{p}\left(T_{1}\right)=9k-15,\;\;W_{p}\left(T_{2}\right)=9k-16,\;\;W_{p}\left(T_{3}\right)=9k-17.$$

**Fig 4:**
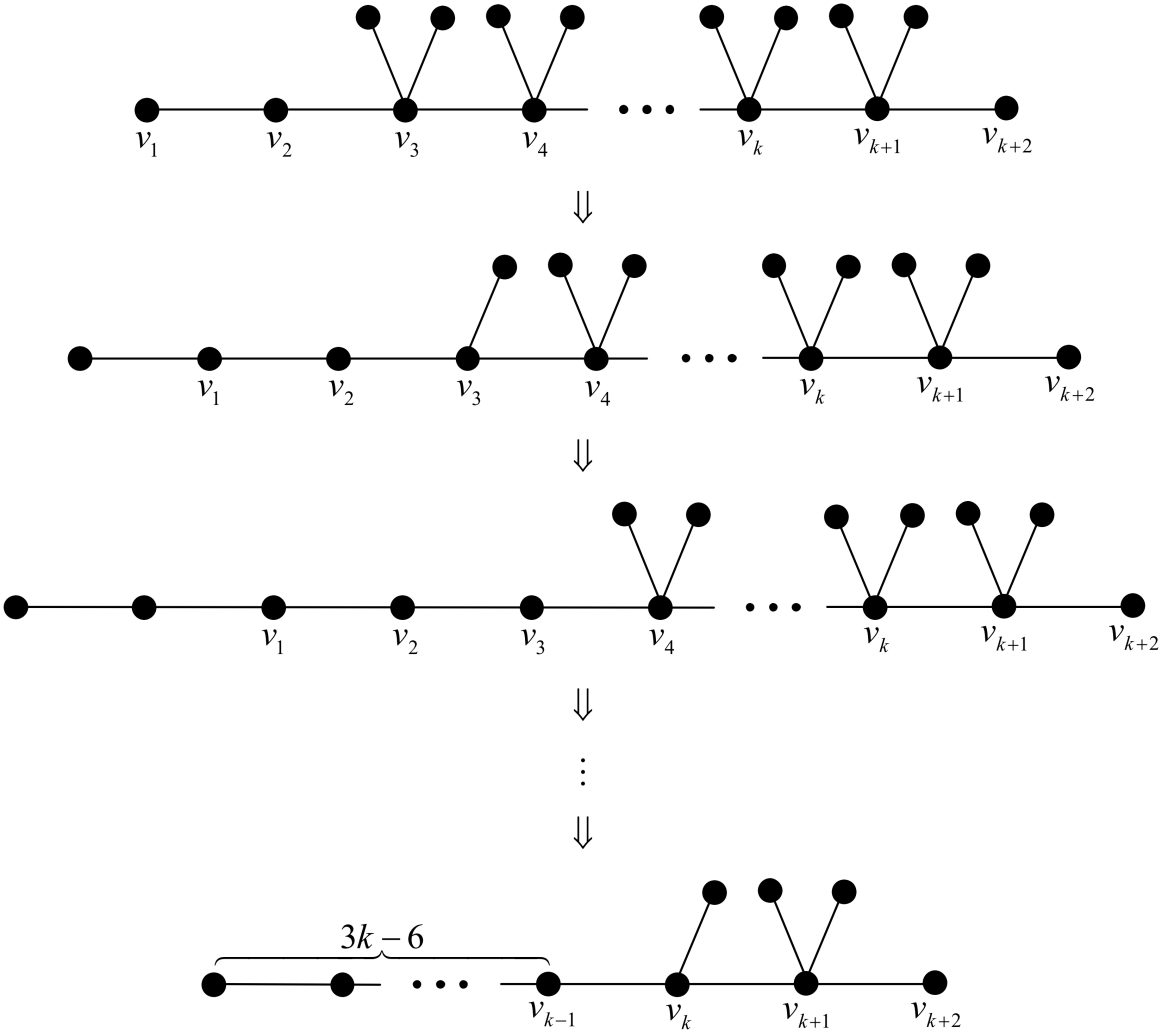
A series of chemical trees of order 3*k* with Wiener polarity indices 9*k* − 15, 9*k* − 18,…,3*k*, respectively.

First, we start with the chemical tree *T*_1_ as depicted in Fig 3, whose Wiener polarity index is 9*k* − 15. We apply grafting pendent path transformations successively at

$$v_{3},v_{3},v_{4},v_{4},\ldots ,v_{k-1},v_{k-1},v_{k},$$

which gives 2*k* − 5 transformations in total. A detailed illustration can be found in Fig 4.

In particular, for the above series of grafting pendent path transformations, by Lemma 2, the Wiener polarity index would decrease 3 each time. This means that we may construct a series of chemical trees of order *n* = 3*k* with Wiener polarity indices

9k-15,9k-18,…,3k+3,3k,

respectively.

**Fig 5:**
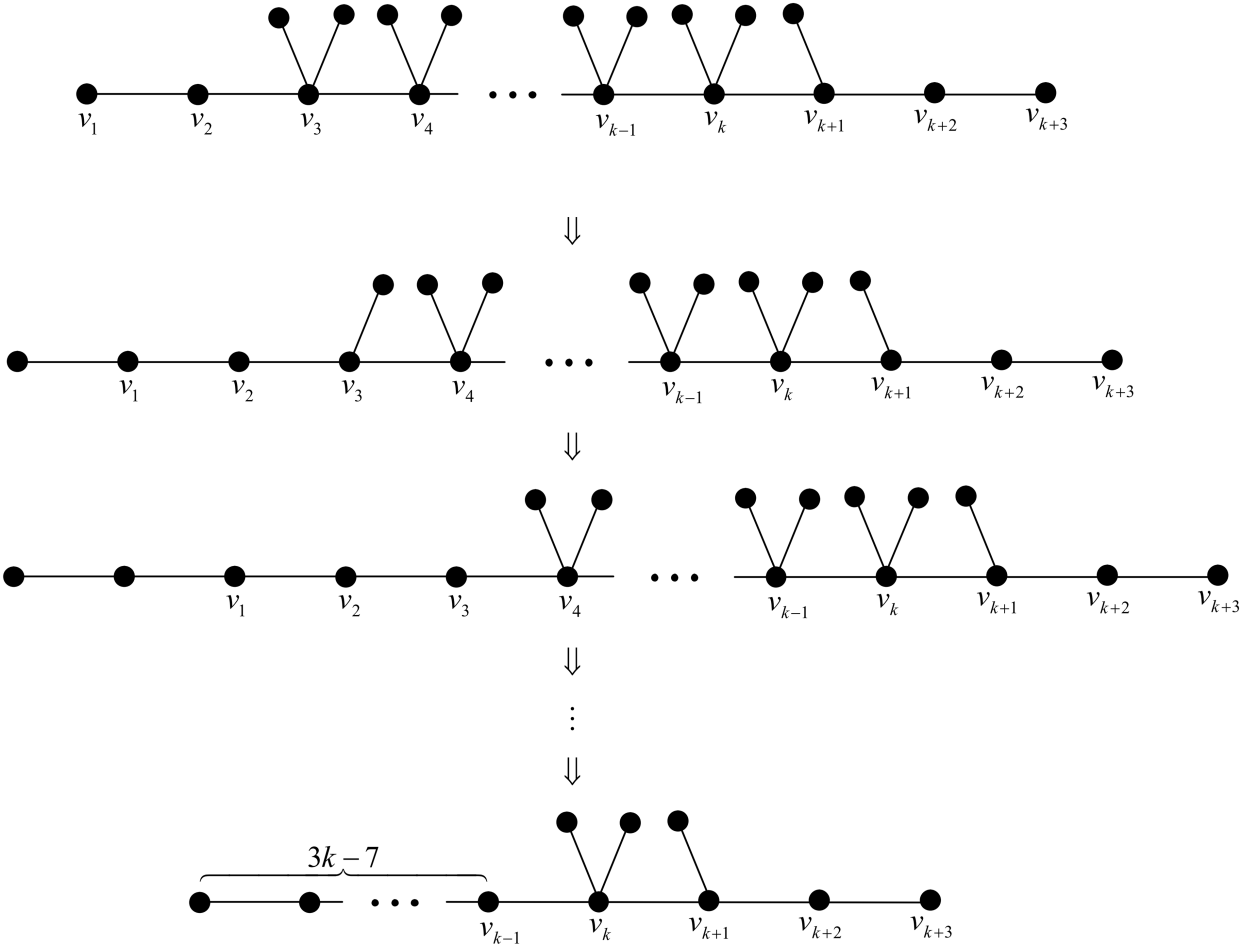
A series of chemical trees of order 3*k* with Wiener polarity indices 9*k* − 16, 9*k* − 19,…,3*k* + 2, respectively.

Next, the initial tree is changed as the chemical tree *T*_2_ in Fig 3, its Wiener polarity index is 9*k* − 16. This time, we will use grafting pendent path transformations successively at

$$v_{3},v_{3},v_{4},v_{4},\ldots ,v_{k-1},v_{k-1},$$

totally 2*k* − 6 times grafting pendent path transformations. The corresponding illustration is shown in Fig 5.

Likewise the Wiener polarity index for the above series of transformations would decrease by 3 each time. Hence we may construct a series of chemical trees of order *n* = 3*k* with Wiener polarity indices

9k-16,9k-19,…,3k+5,3k+2,

respectively.

Finally, choosing the chemical tree *T*_3_ in Fig 3 with Wiener polarity index 9*k* − 17. Similarly, 2*k* − 6 times grafting pendent path transformations will be made, they are successively aimed to

$$v_{4},v_{4},v_{5},v_{5},\ldots ,v_{k},v_{k}.$$

**Fig 6:**
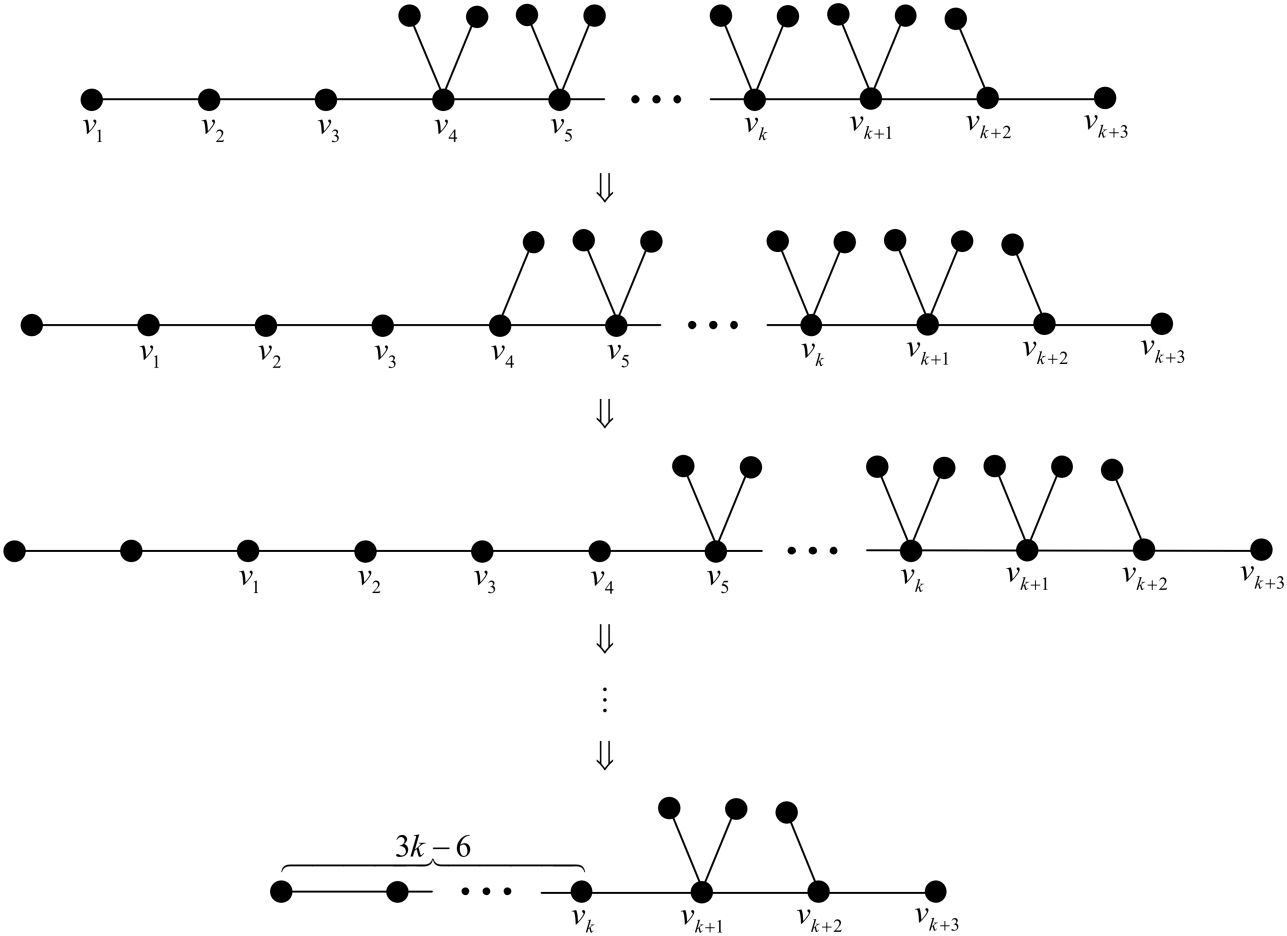
A series of chemical trees of order 3*k* with Wiener polarity indices 9*k* − 17, 9*k* − 20,…,3*k* + 1, respectively.

The process is as seen in Fig 6.

Each time, the Wiener polarity index would decrease by 3. Thus, we may construct a series of chemical trees of order *n* = 3*k* with Wiener polarity indices

9k-17,9k-20,…,3k+4,3k+1,

respectively.

Combining the above arguments, we get a series of chemical trees of order *n* = 3*k* with Wiener polarity indices

3k-3,3k-2,…,9k-16,9k-15,

or equivalently,

n-3,n-2,…,3n-16,3n-15.

Before continuing our proofs for Cases 2 and 3, we first sketch our strategy.

From Case 1, a series of chemical trees of order 3*k* with Wiener polarity indices

3k-3,3k-2,…,9k-16,9k-15

have been constructed. Notice that each of the chemical trees of order 3*k* constructed in Case 1 (see Figs 4, 5 and 6) with Wiener polarity indices

3k-1,3k,…,9k-16,9k-15

has some vertex of degree 4 with two pendent neighbors, say *x*, *y*.

For Case 2, since the order is 3*k* + 1, adding one more pendent vertex to *x* is enough for us to form chemical trees of order 3*k* + 1 with desired Wiener polarity indices. While in Case 3, note that the order is 3*k* + 2, we need to attach a pendent vertex to *x* and a pendent vertex to *y* to obtain our desired chemical trees.

**Case 2**. *n* = 3*k* + 1, where *k* ≥ 3.

In this case, as previous arguments, starting from the chemical trees of order 3*k* with Wiener polarity indices

3k-1,3k,…,9k-16,9k-15,

after adding one more pendent vertex to *x*, from Lemma 3, such operation increases the Wiener polarity index by 3, so we would get a series of chemical trees of order *n* = 3*k* + 1 with Wiener polarity indices

3k+2,3k+3,…,9k-13,9k-12,

or equivalently,

n+1,n+2,…,3n-16,3n-15.

Until now, all the chemical trees with desired Wiener polarity indices are constructed, except when *t* = *n*(= 3*k* + 1). Aimed to this remaining case, we review the chemical tree of order 3*k* constructed in Case 1 with Wiener polarity index 3*k* (i.e., the last chemical tree in Fig 4), obviously it consists of a vertex of degree 2 with pendent neighbor, say *u*. By Lemma 3, attaching a pendent vertex to *u* would increase its Wiener polarity index by 1, i.e., we may construct a chemical tree of order *n* = 3*k* + 1 with Wiener polarity index *n* = 3*k* + 1.

**Case 3**. *n* = 3*k* + 2, where *k* ≥ 3.

Similar to Case 2, we also start from the chemical trees of order 3*k* with Wiener polarity indices

3k-1,3k,…,9k-16,9k-15.

But this time, we need add two more vertices. After attaching a pendent vertex to *x* and a pendent vertex to *y*, and using Lemma 3 twice, this operation increase the Wiener polarity index by 6, which implies that it results in a series of chemical trees of order *n* = 3*k* + 2 with Wiener polarity indices

3k+5,3k+6,…,9k-10,9k-9,

or equivalently,

n+3,n+4,…,3n-16,3n-15.

For the remaining cases *t* = *n*(= 3*k* + 2), *n* + 1(= 3*k* + 3) and *n* + 2(= 3*k* + 4), recall that each of the chemical trees of order 3*k* constructed in Case 1 with Wiener polarity indices 3*k*, 3*k* + 1, 3*k* + 2 (i.e., the last chemical trees in Figs 4, 5 and 6) contains a vertex of degree 2 with pendent neighbor, say *z*. Here by applying Lemma 3 twice, attaching a path on two vertices to *z* would increase the Wiener polarity index by 2. Therefore we may construct three chemical trees of order *n* = 3*k* + 2 with Wiener polarity indices *n* = 3*k* + 2, *n* + 1 = 3*k* + 3 and *n* + 2 = 3*k* + 4, respectively.

The proof is completed.

To illustrate our main result, let us consider an example for *n* = 9.

**Fig 7:**
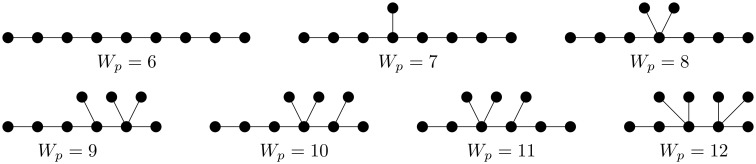
A supporting example for the main result (Theorem 1) when *n* = 9.

**Example 1**. *If n* = 9, *then from* Fig 7, *it is clear that for every integer n* − 3 = 6 ≤ *t* ≤ 12 = 3*n* − 15, *there exists a chemical tree T of order* 9 *such that W_p_*(*T*) = *t*, *and hence*

$$\{W_{p}\left(T\right):T\;is\;a\;chemical\;tree\;of\;order\;9\}=\left\{6,7,\ldots ,12\right\}.$$

## Discussion

In this paper, we prove that the Wiener polarity indices of chemical trees are continuous, that is to say, there is no gap between the minimum value *n* − 3 and the maximum value 3*n* − 15 for the Wiener polarity indices of *n*-vertex chemical trees. As a consequence, we may get a full ordering for the Wiener polarity indices of chemical trees, which extends the ordering about the first three minimum Wiener polarity indices of chemical trees obtained in [21, 22], and the maximum Wiener polarity index of chemical trees obtained in [19, 20].

## References

[1] Trinajstić N. . 2nd revised ed, CRC Press, Boca Raton, Florida, 1992.

[2] Balaban A. T. Chemical graph theory and the Sherlock Holmes principle. 19(1), 107–134 (2013).

[3] Wiener H. Structural determination of paraffin boiling points. . 69, 17–20 (1947). doi: 10.1021/ja01193a005 10.1021/ja01193a005 20291038

[4] Lukovits I. & Linert W. Polarity-numbers of cycle-containing structures. . 38, 715–719 (1998). doi: 10.1021/ci970122j

[5] Hosoya H. & Gao Y. Mathematical and chemical analysis of Wiener’s polarity number in: Rouvray D. H. & King R. B. (Eds.). , Horwood, Chichester, pp. 38–57 (2002).

[6] Miličević A. & Nikolić S. On variable Zagreb indices. 77, 97–101 (2004).

[7] Shafiei F. & Saeidifar A. QSPR study of some physicochemical properties of sulfonamides using topological and quantum chemical indices. . 39(3), 366–373 (2017).

[8] Safari A. & Shafiei F. QSPR models of physicochemical properties of natural amino acids by using topological indices and MLR method. . 39(5), 752–757 (2017).

[9] Chen L., Li T., Liu J., Shi Y. & Wang H. On the Wiener polarity index of lattice networks. 11(12), e0167075 (2016). doi: 10.1371/journal.pone.0167075 27930705 10.1371/journal.pone.0167075 PMC5145185

[10] Hua H. & Das K. C. On the Wiener polarity index of graphs. . 280, 162–167 (2016).

[11] Lei H., Li T., Shi Y. & Wang H. Wiener polarity index and its generalization in trees. . 78, 199–212 (2017).

[12] Ma J., Shi Y., Wang Z. & Yue J. On Wiener polarity index of bicyclic networks. . 6, #19066 (2016). 10.1038/srep19066 PMC4707490 26750820

[13] Ma J., Shi Y. & Yue J. The Wiener polarity index of graph products. . 116, 235–244 (2014).

[14] Zhang Y. & Hu Y. The Nordhaus-Gaddum-type inequality for the Wiener polarity index. . 273, 880–884 (2016).

[15] Du W., Li X. & Shi Y. Algorithms and extremal problem on Wiener polarity index. . 62, 235–244 (2009).

[16] Furtula B., Gutman I. & Ediz S. On the difference of Zagreb indices. . 178, 83–88 (2014). doi: 10.1016/j.dam.2014.06.011

[17] Gutman I., Furtula B. & Elphick C. Three new/old vertex-degree-based topological indices. . 72, 617–632 (2014).

[18] Shafique S. & Ali A. On the reduced second Zagreb index of trees. . 10, 1750084 (2017). doi: 10.1142/S179355711750084X

[19] Deng H. On the extremal Wiener polarity index of chemical trees. . 66, 305–314 (2011).

[20] Du, Z. & Ali, A. The chemical trees with maximum Wiener polarity index. submitted.

[21] Ashrafi A. R. & Ghalavand A. Ordering chemical trees by Wiener polarity index. . 313, 301–312 (2017).

[22] Ali, A., Du, Z. & Ali, M. A note on chemical trees with minimum Wiener polarity index. submitted.

[23] Skvortsova M. I., Baskin I. I., Slovokhotova O. L., Palyulin V. A. & Zefirov N. S. Inverse problem in QSAR/QSPR studies for the case of topological indexes characterizing molecular shape (Kier indices). . 33, 630–634 (1993). doi: 10.1021/ci00014a017

[24] Goldman, D., Istrail, S., Lancia, G., Piccolboni, A. & Walenz, B. Algorithmic strategies in combinatorial chemistry. in: *Proc. 11th ACM-SIAM Sympos. Discrete Algorithms*, pp. 275–284 (2000).

[25] Baskin I. I., Gordeeva E. V., Devdariani R. O., Zefirov N. S., Palyulin V. A. & Stankevich M. I. Methodology for solving the inverse problem of structure-property relationships for the case of topological indexes. 307, 613–617 (1989).

[26] Gordeeva E. V., Molchanova M. S. & Zefirov N. S. General methodology and computer program for the exhaustive restoring of chemical structures by molecular connectivity indexes. Solution of the inverse problem in QSAR/QSPR. . 3, 389–415 (1990). doi: 10.1016/0898-5529(90)90066-H

[27] Skvortsova, M. I., Stankevich, I. V. & Zefirov, N. S. Topological properties of katacondensed benzenoid hydrocarbons: Randić index and its relation to chemical structure. in: *Proceedings of the Conference “Molecular Graphs in Chemical Studies”*; Kalinin State University: Kalinin, USSR, p. 84 (in Russian) (1990).

[28] Zefirov N. S., Palyulin V. A. & Radchenko E. V. Problem of generation of structures with given properties—solution of inverse problem for the case of centric Balaban index. 316, 921–924 (1991).

[29] Skvortsova M. I., Stankevich I. V. & Zefirov N. S. Generation of molecular structures of polycondensed benzenoid hydrocarbons from Randić index. . 33, 416–422 (1992).

[30] Gutman I., Yeh Y. N. & Chen J. C. On the sum of all distances in graphs. . 25, 83–86 (1994).

[31] Knor M., Škrekovski R. & Tepeh A. Mathematical aspects of Wiener index. . 11, 327–352 (2016).

[32] Wagner S. G. A note on the inverse problem for the Wiener index. . 64, 639–646 (2010).

[33] Wagner S. G., Wang H. & Yu G. Molecular graphs and the inverse Wiener index problem. . 157, 1544–1554 (2009). doi: 10.1016/j.dam.2008.06.008

[34] Krnc M. & Škrekovski R. On Wiener inverse interval problem. . 75, 71–80 (2016).

[35] Li X., Li Z. & Wang L. The inverse problems for some topological indices in combinatorial chemistry. . 10, 47–55 (2003). doi: 10.1089/106652703763255660 12676050 10.1089/106652703763255660

[36] Li X., Mao Y. & Gutman I. Inverse problem on the Steiner Wiener index. 38, 83–95 (2018). doi: 10.7151/dmgt.2000

[37] Balaban A. T. Can topological indices transmit information on properties but not on structures? . 19, 651–660 (2005). doi: 10.1007/s10822-005-9010-6 16328856 10.1007/s10822-005-9010-6

[38] Lang R., Li T., Mo D. & Shi Y. A novel method for analyzing inverse problem of topological indices of graphs using competitive agglomeration. . 291, 115–121 (2016).

[39] Gutman I., Togan M., Yurttas A., Cevik A. S. & Cangul I. N. Inverse problem for sigma index. . 79, 491–508 (2018).

